# Perpetrator Perceptions on the Emotions and Motivations Driving Technology-Facilitated Abuse in Relationships: A Story Completion Study

**DOI:** 10.1177/08862605231190340

**Published:** 2023-08-02

**Authors:** Renee Fiolet, Cynthia Brown, Dana McKay, Sally Marsden, Kobi Leins, Bridget Harris

**Affiliations:** 1 Deparment of general Practice and Primary Care, University of Melbourne, Melbourne, VIC, Australia; 2 Centre for Quality and Patient Safety Research, Institute of Health Transformation, Deakin University, Geelong, VIC, Australia; 3 STEM School of Computing Technologies, RMIT, Melbourne, VIC, Australia; 4 Data Ethics, National Australia Bank, Melbourne, VIC, Australia; 5 Monash University, Melbourne, VIC, Australia

**Keywords:** technology-facilitated abuse, relationships, control, abusive behaviors, perpetrators

## Abstract

Technology-facilitated abuse in relationships (TAR) is a widespread social problem that has a significant impact on victim-survivors. Most contemporary evidence on TAR focuses on victim-survivor and practitioner perspectives rather than those of perpetrators who choose to enact this form of harm. Addressing this deficit, this study explored perpetrators’ discourses on emotions and motivations associated with engaging in TAR. Using story completion method, 35 self-identified perpetrators of TAR completed story stems describing scenarios that may precede the use of abusive online behaviors. Reflexive thematic analysis generated three themes. *Abusive behaviors and negative emotions* speaks to maladaptive experiences of anger and/or sadness that can precede a decision to use TAR. *A loss of trust, a desire for control* describes potential motives for using TAR. Finally, *inhibitors of abusive behavior* investigates rationales perpetrators use for avoidance of TAR behaviors, suggesting avenues for working with perpetrators to refrain from using TAR. We conclude by discussing policy, practice, and research recommendations including strategies for technology designers and suggestions for primary prevention and response to TAR.

## Introduction

Intimate partner violence (IPV) and domestic abuse (DA) are human rights violations increasingly recognized as significant contributors to global morbidity and mortality (Devries et al., 2013; Stöckl et al., 2013; World Health Organization, 2013, 2021). All genders experience IPV/DA, however, women are disproportionately impacted with global evidence suggesting that as many as one in three women have survived some form of IPV/DA (World Health Organization, 2021). Many forms of IPV/DA exist, but research has predominantly focused on the physical and sexual behaviors commonly used (World Health Organization, 2013, 2021). Technology-facilitated abuse in relationships (TAR) is a relatively recent pattern of abuse, but one that is widely engaged (Douglas et al., 2019; Woodlock, 2017).

TAR in intimate and familial contexts includes intentional misuse of technology with an intention for coercive control (Dragiewicz et al., 2019) and is frequently referred to as digital coercive control (Harris & Woodlock, 2019), digital dating abuse (Brown & Hegarty, 2018), or cyber dating abuse (Rocha-Silva et al., 2021). Technology can be used to enact other forms of harm, for instance sexual, financial, emotional, or psychological abuse and to facilitate in-person stalking (Harris & Woodlock, 2019). However, there are also distinct acts involving digital media and devices that have been observed and recorded in the literature. Commonly deployed TAR behaviors include—but are not limited to—online stalking and tracking, harassment, and threats made through messaging, checking of mobile phones without permission, doxxing (maliciously sharing identifying material without consent), sharing of personal information and images, destroying or denying access to devices or digital accounts impairing authorized functions or activating unauthorized functions, impersonation, demanding of passwords, and behaviors intended to humiliate, harass, coerce, and control a target (Brown et al., 2021; Woodlock, 2017). Over the last decade, technology has frequently been weaponized within relationships and often intentionally used to isolate a partner from others, and entrap and restrict another person (Douglas et al., 2019). This experience is compounded for marginalized individuals (e.g., migrants and people with disabilities) who are more likely to experience TAR (Afrouz, 2021).

Recent evidence suggests that technology offers perpetrators a unique opportunity to involve others in their enactment of abuse, with social media commonly used to obtain support and momentum from others (Fiolet et al., 2021). Compounding the impact of TAR, others can be galvanized by perpetrators to engage in surveillance or monitoring of a target (Harris & Woodlock, 2019) a behavior known as stalking and noted for its association with fatality (Harris & Woodlock, 2022). While the range and magnitude of TAR impacts are yet to be fully understood, evidence suggests the impact on victim-survivors is significant (Fiolet et al., 2021; Rogers et al., 2022) and includes a range of poor mental health outcomes such as anxiety, sleeping disorders, and depression (Cripps & Stermac, 2018; Woodlock et al., 2020). Impacts may be particularly pernicious among younger people (Brown et al., 2021).

Commonly, those experiencing TAR describe fear as resulting from their TAR victimization experiences (Brown et al., 2021; Dragiewicz et al., 2019; Woodlock, 2017). Service providers working with those subjected to TAR describe the omnipresence experienced by victims of TAR, as inescapable (Fiolet et al., 2021). TAR can be hard for victim-survivors to detect and prove, providing fertile ground for a perpetrator to engage in gaslighting behaviors that can further harm a victim-survivor (Rogers et al., 2022). A victim-survivor’s safety and security can be further undermined through justice system failures to recognize and regulate TAR (Harris & Woodlock, 2022). In efforts to address these issues, research on victim-survivor experiences of TAR and its enduring effects, and advocates for reforms in prevention and response efforts, are increasing (Brown et al., 2021; Cripps & Stermac, 2018; Douglas et al., 2019; Dragiewicz et al., 2019; Fiolet et al., 2021).

Yet to more fully address the issue of TAR and to regulate and prevent its occurrence, an understanding of perpetrator’s perspectives is required. Evidence suggests that empathy, acknowledgment of the impact of IPV, and interventions that stop potential and actual IPV offending are significant in effecting change (Chovanec, 2020). However, factors that influence the enactment of TAR behaviors specifically are yet to be identified. In order to interrupt TAR behavioral patterns and challenge TAR behaviors before they occur, researchers and practitioners need to understand from perpetrators what TAR practices they engage in and what motivates individuals to engage in such behaviors. Insights into the psychological processes that lead to the enactment of TAR are needed to facilitate the development of primary prevention initiatives and guide the work of agencies that deliver perpetrator behavior change programs. Furthermore, an understanding of the tactics, ethos, and drivers of TAR is also vital to advancing “safety by design,” the principles and infrastructure that limit how technology can be co-opted (PenzeyMoog & Slakoff, 2021). Addressing this gap, the aim of this study is to advance the limited knowledge of the emotions and motivations that may influence an individual to engage in TAR, from the perpetrator perspective.

## Methods

A novel approach was taken to exploring the emotions and motivations influencing a decision to use TAR. In recognition of the difficulties associated with engaging perpetrators of abuse, we adopted the story completion method, a research approach that has gained increased popularity when exploring topics of a sensitive nature (Beres et al., 2019; Clarke et al., 2015; Hunt et al., 2018; Kitzinger & Wood, 2019). According to Will et al. (1996), story completion proposes a method of studying respondents’ beliefs and perceptions by inviting them to respond to a story about hypothetical behaviors undertaken by another person. Participants are invited to complete a story presented from a “story stem,” and method that assumes story completions explore participant’s own behaviors from a distance (Clarke et al., 2017; Kitzinger & Wood, 2019). The story completion method is considered particularly beneficial for sensitive topics as the participants are less likely to feel interrogated or defensive about their own behaviors, particularly when the story stem is written in the third person (Clarke et al., 2017; Kitzinger & Wood, 2019). This methodology has been used in research addressing difficult or complex phenomena such as infidelity (Clarke et al., 2015), and the use of pornography (Wood et al., 2017). See Brown et al. (2023) for a more detailed discussion of our application of the story completion method and subsequent methodological learnings.

### Participation

Purposive recruitment was used. Eligibility to participate required participants to be at least 18 years of age, living in Australia, not in a current relationship, and to have not been in a relationship for at least 3 months. Recruitment materials, the Plain Language Statement, and the beginning of the story completion sign-on all indicated that the participant must identify as someone who has used a technical device (such as their phone, computer, and social media) to monitor or harm their partner, or in a way that may have harmed their partner during a previous relationship.

Advertisements were shared with organizations providing relationship programs or treatment programs, and through online social media sites Facebook, Twitter, and Instagram. The advertisements were also featured on websites and social media sites of affiliated organizations that had agreed to share the recruitment material (such as No To Violence, a men’s referral and behavioral change organizations, and Australia’s National Research Organisation for Women’s Safety).

Advertisements included a link to a webpage, which described the purpose and scope of the study. Those who read the introduction and were interested in being involved could click a link to download the Plain Language Statement. Once the Plain Language Statement had been read, participants had the option to read the online Consent Form and consent to the study online.

### Procedure

There were a total of eight story stems created for study participants (see Brown et al. (2023) for a complete list of story stems used). Story stems were developed by a team of experts who have professional or lived experience in various aspects of violence, technology, and/or responding to abuse (including health professionals, a criminologist, technology designers, and abuse and violence scholars). This group included a representative from a victim-survivor of technology facilitated abuse as well as Culturally and Linguistically Diverse and GLBTQI+ representatives.

As the research team were interested in understanding perpetrator perspectives on the motivations and emotions that drive behaviors and decision-making, it was necessary to develop story stems that were specific enough to create an opportunity for the participants to choose TAR behaviors within a scenario but was open enough to allow the participant to also choose other or non-abusive behaviors and explain the reasoning for the account they outlined. This structure was created to avoid influencing participants toward proposing any specific action.

Stories deliberately used the same two names (Lee, Alex or Sam, Ash) that were considered gender neutral, to minimize participants assumptions about gender of the characters, or the nature of the relationship. All story stems provided a brief scenario and then asked the participants to complete the story in at least 250 characters. An example of a story stem follows:

Lee and Alex are having trouble in their relationship, and Lee has been thinking about it a lot. Lee is awake and worrying about the relationship, Alex is upstairs asleep. Lee notices that Alex’s phone and laptop are unlocked on the kitchen table and. . .. (please write about what Lee thinks, does and feels, and why Lee chooses that course of action).

Members of the research team circulated a link to the story stems and a feedback survey via departmental newsletters and via Facebook. The story stems were subsequently anonymously piloted by 12 colleagues and associates of the researchers. The feedback survey was provided to everyone who piloted the story stems so that they could provide feedback to the research team about suggestions/amendments. These were circulated with the story stems in departmental newsletters at the three universities at which the authors were based.

Participants who completed the Consent Form were then asked to create a unique identifier which could be used later to validate their identity as a participant (last two letters of the street they live on, last digit of the year of their birth, and last three digits of their mobile phone number). After establishing their unique identifier, they were directed to a Qualtrics survey (hosted by the University of Melbourne), which posed demographic questions prior to offering participants the opportunity to answer two story stems. Every participant first completed story stem one and then completed one of the remaining seven randomly allocated story stems. This elicited data relating to multiple scenarios and contexts in which TAR it may occur. The randomly allocated story stems were each completed by four or five participants.

Following completion of both stories, participants were asked to provide their email address (which was used to issue a twenty-dollar gift honorarium). Once the email address had been provided, the research team was able to ask the participant to verify their unique code. If a genuine unique code was provided, then the gift voucher was emailed back to the participants, and the research team immediately deleted the email address to reduce future risk of identification. This was one of several ethical actions taken by the research team to protect those involved in the study.

### Ethical Implications

The research team had an ethical obligation to protect both those involved in the study as well as those intended to benefit from the research. The following actions were undertaken to meet this obligation.

#### Protecting Victims-Survivors

We restricted participants to people who had not been in a relationship for at least 3 months to mitigate the risk of post separation/retaliatory abuse occurring to ex-partners.

#### Protecting Researchers

A study-specific website and email address were set up to protect the researchers from harassment. Online methods reduced any physical risk to researchers (Westmarland & Bows, 2018).

#### Protecting Perpetrator Participants

The story completion method ensured that people were not disclosing their own (potentially criminal) behaviors. Participants created a unique user ID so that their participation could be verified while their email address was deleted by the research team. All data were password protected and held on a secure server. Participants were provided with a list of supportive resources (e.g., Lifeline and Relationships Australia to address psychological needs and educational opportunities) in the Plain Language Statement and at the completion of the online exercise.

Ethical approval was provided by the University of Melbourne—Ethics ID #20939.

### Data Analysis

Inductive reflexive thematic analysis was employed in accordance with Braun and Clarke’s (2006) method for thematic analysis. The research team was made up of a diverse group of researchers from a variety of backgrounds (health, psychology, technology, data ethics, and criminology) who had different ways of thinking and identified with various philosophical and theoretical assumptions; this rich diversity was prioritized during analysis and led to robust dialog at multiple times throughout the analysis process. The research team all identify as women, all but one identifies as heterosexual and some of the team have lived experience of TAR. Conscious of how our expertise and experiences shaped our reading of data during the review process, we compared and contrasted codes and themes and collectively reflected on how these factors might have influenced our analysis. Then, we worked together to develop and define a thematic structure of descriptive and interpretive codes. This strengthened trustworthiness of the analysis.

Data analysis began with all members of the research team familiarizing themselves with the story completion data by reading the answers, both individually and then as a group for discussion of initial codes using inductive coding. Following the identification of initial coding, the research team worked together to group the codes. Clustering of codes led to the identification of four dominant themes. These themes were then discussed by the entire research team over two meetings and narrowed down to three main themes, which were endorsed and named. As a final measure, our analysis and the meaning of the themes to current practice, policy, and research were shared with key stakeholders in TAR research and industry specialists.

## Results

Although a total of 42 people completed the story stem exercise, seven did not provide their email address nor unique identifier when prompted so their data was not analyzed. Thus, analysis was completed for data provided by 35 people over 5 months (with each participant completing two stories, totaling 70 stories). The participants demonstrated a reasonable level of diversity. Of the 35 participants, 21 identified as men, 12 as women, 1 identified as a trans man, and 1 person identified as non-binary. Twenty respondents were aged between 26 and 35 years, with 18 and 25 years (*n* = 13) the next most common cohort. Just two respondents were aged between 36 and 45 years of age. Most participants were heterosexual; however, two of the participants identified as being gay, two characterized themselves as being bisexual, and one stated that they were pansexual. Most participants lived alone, and all but two were born in Australia. Educational attainment was reasonably high with only one person not completing secondary school. One-third of participants had a Bachelor or Postgraduate degree, whereas one-third were in trade.

Although participants were asked to finish the character’s stories, some referred to the (potentially abusive character) in the first person, using “I” or “my” instead of using the character names. Other participants began their stories by using character names but ended their stories using first person. In 38 stories, the putative abusive partner was referred to in the first person, and in a further four stories, they were not referred to with any gender; the putative victim was not gendered in 40 stories. In 22 stories, where participants used gendered pronouns, these were applied to the putative perpetrator and gendered pronouns were also given to the partner (victim) in the story. For victims, the mismatch occurred in eight cases, and all but two were straight women. The remaining two were straight men who described a gay relationship in the Alex and Lee scenario. While participants were encouraged to write stories of minimum 250 characters in length, the average story was 407 characters in length.

Thematic analysis generated three overarching, harmonious themes. *Abusive behaviors and negative emotions* speaks to the anger and/or sadness that often precedes a decision to engage in TAR. *A loss of trust, a desire for control* in the relationship describes potential motivations for the use of TAR. Finally, *Inhibitors of abusive behaviors* explores some of the rationales perpetrators use to refrain from engaging in TAR.

### Abusive Behaviors and Negative Emotions

Our findings suggest that there are multiple emotions that influence the choice to use technology in abusive ways. The most frequently mentioned emotions were anger, fear, suspicion of betrayal, and sadness.

Most participants identified anger as a strong emotion present in their narrative decision-making about technology-facilitated behaviors. The anger tended to vary in intensity from “feeling the anger build” to extreme anger “Sam instantly felt that he was going to die of anger.” The anger was often in response to perceptions/ideas that their story partner had deceived them in some way “Alex was very angry at the harm Lee had done to him, and planned to wake up and have a showdown with Lee.” Others felt anger in response to actual evidence their character had deceived them “he was very angry when he saw the photos of his ambiguous relationship with others” and “after seeing it, he is very angry.” Some respondents indicated that anger would lead to an altercation “Lee immediately took the phone and went upstairs angrily to ask Alex.”

There were also examples of perception of indiscretions sparking anger:

Lee was very curious, who would send a message to Alex at this late hour? He went to the kitchen and picked up his phone. It was discovered that it was a message sent by a strange man, and the content of the chat between the two was very ambiguous, which made Lee very angry. He felt betrayed.

Any of the stories that indicated there had been a separation or there was a potential problem in the relationship tended to elicit the most emotional responses. In particular, the story that suggested shared images should be deleted elicited strong responses from most of the participants who expressed that they would not delete the images and would instead keep them. Fear and suspicion of betrayal were commonly noted to motivate the use technology-facilitated negative behaviors.

Lee sees an opportunity to evade Alex’s privacy and understand more about what she is thinking about. The curiosity for people can be overwhelming, and for Lee who seems to be overthinking their situation, snooping is an easy solution to help him affirm his concerns or suspicions. Lee then opens her phone and laptop and proceeds to search through social media platforms and potential notifications that could provide him with any information of how Alex is feeling or what she has been thinking about lately. This could be targeted in order to match and affirm his suspicions by looking for any potential individuals that could be the cause of their conflict.

 Some respondents admitted that suspicion was driven by fear; “was afraid Ash would lie to me about her movements.” These sentiments tended to be influenced by personal insecurity “I feel insecure in a relationship,” some of which appeared to be a long-standing issue “My childhood environment has made me feel inferior.” These emotions caused the story-writer to doubt the partner and the relationship “I am afraid of what she is hiding from me. I am afraid that she is in love with others at the same time.” Fears and insecurities informed decisions to use TAR behaviors on many occasions:

I’ll install a GPS app when I set up Ash’s phone. I’m trying to track Ash’s daily movements using GPS software. It gives me a good sense of security. I was afraid. . .Although I know it is wrong to do so, I am a person who lacks confidence.

There were instances when such anxieties also motivated the participant to write about a search for “evidence” that the character’s partner could be losing interest in them:

Lee could also be searching through her conversations with her close friends to understand what Alex is thinking of Lee at the time.

Other stories suggested that fear could drive the investigation for negative conversation about themselves rather than information about the partner:

Lee will first look at the SMS, social media and other chat records in his mobile phone to see if there is any information unfavourable to him.

When there was some hesitancy in the mind of the story-writer, panic and insecurity were said to justify and provide rationale for searching a partner’s technology:

Lee checks that Alex is still asleep then takes the phone into the toilet so that Alex will not see if she comes down and searches through it. It will start with messages in social media and checking emails. Although Lee will feel some guilt in doing this it will be better than always wondering if Alex is cheating. When Lee finishes checking the phone for all the different apps that Alex can message on, the phone is returned and Lee takes the laptop in. This time Lee searches through all of the folders on the laptop that are named in a weird way to see if Alex has been keeping any photos of someone else. Lee finds what he has been suspecting all along and is going to confront Alex about it.

Some of the stories failed to indicate whether the fear was derived from personal doubts or a general sense of mistrust in their partner. On one hand, some participants appeared not to connect suspicions to this worry at all. Indeed, one gave an example where the actor only needed to lack an understanding of their partner’s activities to be doubtful “If I don’t understand my partner’s social activities, I will be suspicious.” Others cited specific activities as a reason for the character’s distrust:

Ash is curious to understand why Sam is now spending more, this could be in the form of checking her expenses and bank app, to try and understand whether her income has increased and if she is receiving income from a new source or new person.

Doubts tended to grow and become exaggerated “What is Alex hiding? Running up the stairs Lee’s mind is going into overdrive imagining all of the things Alex is probably getting up to.” Some story characters quickly judged the story partner’s behaviors “I can’t accept being lied to and betrayed.” This occurred even if there was no actual evidence of unscrupulous activity “because Ash has not contacted me for 2 h, I would guess Ash will have another partner. I will also look for discussions about partner cheating.”

Another frequently cited emotion in the stories was sadness. The sorrow spoken about tended to be in relation to perceptions that a partner had betrayed the character, rather than sadness causing inappropriate behaviors or responses. Thus, the melancholy described was different to the emotions of anger, fear, and suspicion. Some of the described sadness was quite deep and suggested a break-down in relationships to have a significant impact “he was sad and broken,” yet some acknowledging it would be necessary “Sam is so sad that he decides to break up with Ash.”

However, distress tended to be associated with perceptions that an ex-partner was moving on with someone else “Ash and his new partner’s smiles filled with happiness deeply hurt Sam’s heart” rather than the demise of their own relationship.

While responses to most story stems provided plentiful evidence to generate the theme of abusive behaviors and negative emotions, the story stem that was generated about home automation/smart home technology provided little evidence of either abusive behaviors or negative emotions. This may be due to this particular TAR context being associated with different behaviors and motivating emotions when compared with other forms of TAR, or to home automation systems being less common than some other technologies such as mobile phones and laptops thus participant’s prior experiences or exposure to discourses about the harmful use of automation systems, being less salient.

### A Loss of Trust, A Desire for Control

Half of the participants identified a loss of trust or concerns that their relationship was in trouble as driving the choice to use TAR behaviors. There were times when the motivation to address betrayal was also provided as justification for the act “Although Lee will feel some guilt in doing this it will be better than always wondering if Alex is cheating.”

Few participants specified a reason for losing trust; a reason for questioning the story partner’s loyalty was not always present “my lack of trust in my partner.” Some responses indicated that any tension in the relationship could generate questions about their partner’s faithfulness “Lee feels like where there is trouble there is probably cheating and wants to investigate if this is the case.”

Where there were reasons cited for such uncertainties, the actions that triggered those concerns could have been easily explained by the partner; however, the story character formed their own conclusions:

Lee opens up Alex’s phone and tries to use Alex’s password that has been used forever but it doesn’t work! Feeling the anger build, Lee needs to know why Alex would change their password now after all of this time.

There were some examples where participants’ narratives suggested that the figure’s internal dialog hastened and heightened the doubt:

Gets onto messenger to try and contact Ash. Sam has tried ringing Ash four times and has sent some messages but is not getting a response. There is no response on messenger either, so Sam then has a look at Ash’s friends Facebook and Instagram profiles to see if they are posting pictures from their nights. Starting to feel like he wished that he had Ash’s friends’ numbers or was connected to them on snapchat so he could find out where the hell they all are and why no-one is responding. Thinking the worst might be happening. . .

Through their stories some participants identified the lack of trust as an internal/gut feeling “my feeling that she might have betrayed me.” However, there were examples where participants were not relying on those feelings, instead choosing to exercise reservations because of broader troubles in the relationship “it has been more complicated in their relationship lately that he feels the only way is to go behind her back and do some research for himself.” There were also several instances where the two were linked “I’m more interested in whether the difficulties in our relationship have anything to do with Alex’s social activities.”

In their stories, some participants created accounts where their characters went to considerable length to deceptively monitor the activities of their partner while trying to avoid future problems in the relationship. Reference to concealment attempts suggests awareness that the behaviors they depicted were in some way undesirable:

Sam writes down all of the passwords so he can check that Ash doesn’t change them later on and then adds an app he heard about called Life360 so he can see where Ash is all of the time. Worried Ash might find the app, he hides it in with a heap of exercise apps that he also installs. He then remembers that he can locate Ash if he installs a running app he is already using so he downloads that too in case the live360 doesn’t work well.

There were instances when the participant expressed an explicit desire for control in their relationship “so that Sam can control what Ash does online at any time.” A few respondents were explicit in their discussion about control:

In a relationship, I often check my partner’s cell phone information. I think this will bring me satisfaction. I don’t want my partner to conceal me and deceive me. I want to master their social relationships. So for my partner’s mobile phone and laptop, I will open it, and I will open it often.

If I were Lee, I will check Alex’s private information. I am a controlling person.

Others attempted to explain their character’s motivation: “It gives me a good sense of security.” There were notable examples of narratives demonstrating an implicit intent for the perpetrating character to gain power through substantiation of their suspicions “snooping is an easy solution to help him affirm his concerns or suspicions.” A further example demonstrated that the participant, in their narrative, was quite certain that the actor was correct in distrusting their partner “I will go through her phone and laptop to verify my suspicions.”

There were few instances when participants spoke about payback or revenge for a perceived transgression; however, when they spoke about taking action, intentional harm of the fictional person was suggested:

. . .he copied all the photos and transmitted them to the social network. At the same time, he kept looking for all kinds of information in Alex’s computer to see if there were any harmful videos. . .he copied them and kept them as evidence.

Some actions taken by the characters indicated that there was not an inherent intent to harm; rather, the character wanted to do something that benefited themselves and did not appear to understand impact it may have on the other person in the relationship. One story stem was about an ex-partner asking the story-writer to delete an old video they had made together and the completed story indicated that this wish would not be respected, citing their right to retain the digital material “if Ash asks me to delete the video, I won’t agree. I will save each of my relationships and photos and videos. This is my memory, I will not delete videos and photos just because we broke up.”

Straight women commonly reported choosing not to perpetrate. When women write about women committing abuse (using either “I” or “she” pronouns for abusers), it is common, though not universal to describe insecurity, rather than a desire for control.

### Inhibitors of Abusive Behavior

Although there were many accounts where characters pursued TAR, there were examples where fictional perpetrators recognized that such harms complicated relationships, upset partners, and fostered mistrust. This realization caused some to reconsider using TAR “I might check Alex’s cell phone and laptop, but sensibly tell me that I can’t do this. If I do this, my relationship with Alex will be more difficult to reconcile.”

Awareness of better ways to manage the proposed scenarios were evident:

It’s better for me to hear the truth from Alex rather than reading messages illegally. I know it would be very tempting to have a look at phone and laptop, [but]it will exacerbate the relationship. Who knows if we can make it better if I don’t violate Alex’s privacy?

There was also suggestion that some valued the foundational nature of trust in a relationship “I respect the privacy of my partner. I think that in a relationship, partners need to trust each other. There must also be free space.”

On occasion, this awareness translated to a behavioral change: “The moment I grab Ash’s phone, I know it is not right to do so.” Such awareness was at times related to reflections on previous experiences of guilt:

Lee has only looked at a phone of her partner once, when Alex was sleeping a few months ago. She felt so guilty and breathless she put it back.

Some data suggest that maturity or life-experience may correlate with a belief that TAR constituted inappropriate relationship behavior:

He realises this was something he would have done when he was a bit more immature and inexperienced with relationships. Now, upon thinking about it more thoroughly of how he values and respects Alex’s privacy, he understands that that’s not the right thing to do by Alex and their relationship. He started to make some mental note about what could potentially be the thing in their relationship that he’s worried of, and was thinking of brainstorming of the ways to go about approaching this. Maybe if he wasn’t feeling confident enough of dealing with this on his own, before having a conversation with his partner, he would consider talking to his GP to get a referral to see a counsellor, either for himself or for their relationship.

Despite these examples, few narratives described genuine respect for partner privacy in their stories, while some expressed caring about the story partner’s level of comfort “It can also make your partner uncomfortable. I’m not going through Alex’s phone or laptop” or happiness:

If I were Lee, I would not check Alex’s privacy. If Alex knew that I checked her privacy, she would feel uncomfortable and unhappy.

## Discussion

Our research found TAR is used across a range of genders and sexual identities. Based on the narratives it appears that perpetrators who weaponize technology to harm their partners can feel anger, suspicion, fear, or sadness when enacting these harms. We identified that mistrust and a “need for control” over the relationship are viewed as strong motivators for the choice to use TAR. These motivations have similarities to the paranoia identified by many when speaking about emotions such as fear. However, we also found that some people have the capacity to reflect on their behaviors and consider refraining from TAR to avoid relationship damage and/or resolution.

The anger portrayed though the narratives supports the findings of Alsawalqa and Alrawashdeh (2022) whose research among 47 male Jordanize university students revealed that most participants identified anger as the primary emotion influencing their TAR behaviors. Although culturally different samples, research from Jordan and now Australia both recognize the role of anger in motivating perpetration of TAR (Alsawalqa & Alrawashdeh, 2022). Suspicion was also a common feature in the findings of both studies “I felt that there was a sudden change in her behavior with me, her feelings, interest. . .Certainly there was someone else” (Alsawalqa & Alrawashdeh, 2022, p. 11). Notably, the participants in the Jordan-based study expressed their desire for revenge more strongly “. . .it increased my need for revenge. Yes, I deliberately wrote the sarcastic and insulting phrase and wrote ugly words on her photos and posted them on her social media site for her, on a daily basis. . .” than in the current Australian study.

Our findings on anger contrasted recent evidence emerging from Aotearoa (New Zealand) where a study undertaken among adults who use technology found that the majority of those engaging in TAR (11% of the studied population) cited humor; explaining that their behavior was “a joke” (Pacheco & Melhuish, 2021). It is important to note that this study was not focused on a perpetrator cohort, and that the measure used did not refer to anger as one of the behavioral motivators. The closest category to anger was “to get revenge” (Pacheco & Melhuish, 2021) of which only 4% of participants identified as a motivator for engaging in TAR.

The findings related to using TAR—specifically to monitor or stalk a partner because of suspicion, mistrust, and fear of losing control—are similar to survivor perspectives on motivations (Dragiewicz et al., 2019; Havard & Lefevre, 2020; Henry et al., 2022). They also support Chan and colleagues’ conclusions that individuals with greater emotional dysregulation are more likely to perpetrate online stalking behaviors (Chan et al., 2022). Emotional dysregulation has been identified as a predictor of offline stalking behavior (Chan et al., 2022) and is known to be associated with higher rates of physical violence (McEwan et al., 2009). Cavezza and McEwan (2014) concluded that there is little difference between the characteristics and motivations of offline stalkers to that of individuals who use technology-facilitated stalking, and suggest that the same assessments, treatments, and judgments be used when responding to both forms of stalking perpetration. Our findings suggest that emotions, such as anger, can escalate quickly. Given technology-facilitated stalking (which our findings suggest can stem from anger) has been associated with fatal violence in death review cases (Harris & Woodlock, 2022), we concur that those using TAR should be approached with the same caution and disciplinary measures as those using more traditional stalking behaviors.

Technology-facilitated monitoring of a partner’s whereabouts and/or activities (offline and online) have also become relatively normalized (Brown et al., 2022; Harris & Woodlock, 2019). Harris and Woodlock (2019) recommended society denounces TAR to render it socially unacceptable in ways similar to physical and sexual violence. This may be particularly important among teenagers (Lucero et al., 2014) and young people (Brown et al., 2022). The current findings offer new insights into perpetrators’ potential justifications for engaging in TAR; insights that could be used to inform such a denouncement. For example, some story characters demonstrated a capacity to contemplate the impact of TAR on victim-survivors. Along with the behavioral motivation findings, this furnishes design opportunities for primary prevention initiatives, potentially enabling more targeted education and bystander programs for delivery to individuals who may engage in using TAR.

These findings also highlight opportunities to disrupt and prevent further perpetration by those actively engaging in technology-facilitated abuse. Understanding perpetrator tactics and strategies can assist technology developers in designing or regulating technologies in ways that mitigate or reduce TAR behaviors. This could include the development of technological mechanisms that interrupt a user when they demonstrate online signs of anger or sadness, in turn preventing their engagement in TAR. Technology developers could also explore the potential of “persuasive design” mechanisms that seek to promote prosocial or healthful behaviors, for example recognizing escalation of emotions and suggesting healthy behaviors such as stepping away from the digital device or speaking with friends (Baumeister et al., 2023; Purpura et al., 2011; Toscos et al., 2006). Thus, for policymakers, practitioners, and industry alike, the findings from the current study offer important insights for use to inform, create, and reform work in this sphere.

From a theoretical perspective, negative peer support (that is hostile to a particular cohort) accomplished through guidance from friends or peers who perpetrate violence, can encourage, justify or espouse violence, and increase the likelihood of victimization (Dekeseredy et al., 2019). Conversely, prosocial peer supports and connectedness, including through the use of technologies, is theorized to reduce victimization (Marganski et al., 2022). The influential role of negative and positive peer support predicates the importance of the current findings and highlights the potential opportunity to interrupt behavioral patterns when signs of imminent TAR perpetration are present. This may be particularly relevant among young people (Dekeseredy et al., 2019) who may be more receptive to prevention messaging than adults. Alternative solutions may be needed to interrupt the behavioral patterns and perpetration of TAR among adults, and further research is required to verify this. There are multiple policy, practice, and research recommendations and implications related to our research findings discussed below.

### Policy Recommendations and Implications

Technology occupies key roles in our lives, enabling and providing opportunities for education and work; leisure, social, and civic activities and engagement; and management of lifestyle and households (Douglas et al., 2019). Digital devices also provide opportunities to facilitate violence, marginalization, discrimination, and exclusion (Harris & Woodlock, 2019). This harmful use of digital devices threatens technology’s benefits and pathways to freedoms and rights enjoyed by many (Douglas et al., 2019) and harms individuals (Harris & Woodlock, 2019). Initiatives and policies that address and prevent TAR are lacking despite victim-survivors, advocates, and practitioners calls to urgently address these harms. In order to educate society and prevent the occurrence of TAR, awareness and primary prevention campaigns are needed. Expanding education, such as the Respectful Relationships programs presented in many Australian schools (Pfitzner et al., 2022), campaigns could focus on “healthy” relationship behaviors, premised in respect and consent. Campaigns could also address the normalization of TAR, and aid in shifting privacy boundaries, which enable TAR.

Policies and practices that seek to address, prevent, and regulate TAR are also needed. Recognition that the features, functions, and management of technologies can at times contribute to or enable TAR is central to developing principles and infrastructure that limit the harmful use of technology. In other words, investment in efforts to minimize or stop harm through safety by design approaches is needed (PenzeyMoog & Slakoff, 2021). So too are technology-mediated mechanisms that interrupt the potentially harmful behavioral patterns of perpetrators.

### Practice Recommendations and Implications

Responses to TAR also need to be enhanced. By identifying TAR perpetrator rationales, explanations, and motivations, strategies to interrupt and challenge these can be incorporated into perpetrator behavior change programs. Primary prevention is required, and we suggest that this begins at an early age when respectful relationships are being examined within the school environment.

From a technology development perspective, there is potential to intervene when technology users are showing online (and offline!) signs of heightened anger or maladaptive responses to sadness and anger. Although some would argue that it would be unethical to influence an individual’s behavior in response to actions or signs that emotions were escalating, there is a need to weigh this against the questionable actions tech companies currently take regarding product advertisement. If there is enough knowledge and tolerance to allow technologies to tune into personal conversations and provide advertisements that are geared toward our current moods/practices and discussions, there must be capacity to design technologies that would respond to maladaptive emotional responses.

In providing health services and support for people who have perpetrated TAR, we can reflect on the findings of how emotion played out in the story stem completions. Sadness and anger were among those disclosed, and health assessments and services could assist in techniques and approaches to manage emotional regulation and mental health. Speaking with people who have used TAR about identifying and regulating their emotions and employing strategies to avoid the use of harmful behaviors may prove to be effective. Additionally, findings from the current research can be used in prevention work and behavior change programs for encouraging attendees to consider how their own emotional states may influence a decision to make poor choices regarding online behaviors.

### Research Recommendations and Implications

To date, research insight into TAR perpetration is scant. Predominately, scholarship has involved quantitative self-reporting surveys of “digital dating abuse,” involving university/college or high school students, often cohorts from the United States (Bennett et al., 2011; Lindsay et al., 2016; Reed et al., 2016). Brown and Hegarty (2018), on reviewing the international literature, found highly variable perpetration rates (3%–94%). This disparity is due, in part, to highly variable definitions of both technology and TAR (Brown & Hegarty, 2018).

While useful information can be gleaned from quantitative research, there can be several limitations with such studies. Specific behaviors are identified and tested by researchers, but context—the intent and effect of behaviors and relational dynamics—may be missed (Dragiewicz et al., 2019). This is problematic because it is not merely an act but the intent and impact and meaning that must be examined (Brown & Hegarty, 2018; Brown et al., 2021). A survey may, for example, test for when respondents have accessed a partner’s device; however, this may be done with the consent of the other party. Alternatively, it may, as some respondents in this study show, be performed for the purposes of surveillance, or control, possibly by an abusive partner. The context of each example is starkly different but may not be detected in a survey. Moreover, surveys rely on self-reporting of behaviors, though scholars contend that those victimized may “over” explain their behaviors (such as resistance to violence, self-defense, or protective violence) and perpetrators tend to under-explain or minimize, deny, excuse, or justify their behaviors (Bancroft, 2003; Harris & Woodlock, 2019).

In qualitative research, the aforementioned tactics of perpetrators (to minimize, deny, excuse, and justify) provide insight into how and why violence is enacted and indeed perpetrators may not identify or classify their behaviors as violence. Such research with perpetrators is classed as high-risk, including because it may involve the disclosure of criminal activity. Social undesirability and shame can be also deterrent to perpetrators opting in to research or disclosing more generally (Hashimoto et al., 2021), yet despite these barriers further research with perpetrators is needed to provide an evidence base to inform and guide primary prevention and awareness campaigns. This evidence would also provide opportunities to interrupt and address harmful behavior, including in behavior change programs. Therefore, more research using unique methods that encourage perpetrators to disclose their abuse and their reasons for using TAR is required.

## Strengths and Limitations

Strengths of this study emerge from the original application of the story completion method to explore TAR and its perpetration, in an Australian context. As there is increasing interest in story completion methods in Australian research, this study provided learnings for other qualitative researchers. Because the methods have been applied to this relatively new form of abuse, we were able to generate data that may not have been realized had other methods been used. A further strength was the diversity of the participants, including those who identified as men, women, trans man, and non-binary, and heterosexual, gay, bisexual, and pansexual identities.

The authors identify limitations in the generalizability of the findings to the broader Australian audience for three core reasons. First, all but two participants were born in Australia, which is not representative of the Australian population of whom up to 30% are born overseas. Additionally, although participants spanned a range of genders and sexual identities, those diverse representations were not numerous enough to identify findings specific relating to diversity. Third, the majority of participants were between the ages of 26 and 35 years, which fails to represent the large number of young Australians who engage in TAR (Office of the eSafety Commissioner, 2017). Another limitation of this study is the small number of participants who took part. In using a novel form of engagement, we conclude that there is still a lot to learn about how to engage perpetrators of abuse—a hard to reach population due to the social undesirability of their behaviors and the risk of self-incrimination. Despite the challenges of recruiting participants to engage through the story completion method, we believe there is potential to advance the use of this method in the IPV/DA and TAR fields. A more detailed discussion of these challenges and opportunities of using the story completion method to conduct research among TAR perpetrators is available in an additional paper (Brown et al., 2023).

The most significant limitation to this study is the fact that story completion is a method used to explore respondents’ beliefs by inviting them to reply to a story about hypothetical actions undertaken by a fictional character so they are delving into behaviors from a distance; caution must be taken in interpreting the findings as representative of perpetrators actual emotions and behaviors.

## Conclusions

Previous research on TAR has focused on victim-survivor and service provider perspectives and experiences. Although valuable in informing responses to TAR, evidence regarding how to intervene and prevent TAR from occurring needs to be informed by knowledge of those using the behaviors. This study used a novel approach to explore perpetrator perspectives and despite the limitations of our research, found that those using TAR identify experiences of maladaptive anger and/or sadness as emotions that may inform the use of TAR. Project findings add new and relevant knowledge to existing evidence, with perpetrator narratives suggesting that TAR can be motivated by a lack of trust and desire for control, demonstrating that online forms of harm may be driven by the same motivators as other forms of IPV/DA. This evidence could contribute to attitude and behavioral change if used to inform primary prevention efforts and mandatory and voluntary programs targeting perpetrators of violence. Our findings provide a significant contribution for technology developers who can use these insights to inform designs that deter perpetrators from using harmful online behaviors. We have made multiple recommendations for policy and practice, yet it is the recommendation that further research is undertaken to explore perpetrator perspectives more thoroughly that requires the greatest attention.

## Supplemental Material

sj-docx-1-jiv-10.1177_08862605231190340 – Supplemental material for Perpetrator Perceptions on the Emotions and Motivations Driving Technology-Facilitated Abuse in Relationships: A Story Completion Study Click here for additional data file. Supplemental material, sj-docx-1-jiv-10.1177_08862605231190340 for Perpetrator Perceptions on the Emotions and Motivations Driving Technology-Facilitated Abuse in Relationships: A Story Completion Study by Renee Fiolet, Cynthia Brown, Dana McKay, Sally Marsden, Kobi Leins and Bridget Harris in Journal of Interpersonal Violence
