# Supplementary material for: Perpetrator Perceptions on the Emotions and Motivations Driving Technology-Facilitated Abuse in Relationships: A Story Completion Study
Source: J Interpers Violence. 2023 Aug 2;38(23-24):11999–2024. doi: 10.1177/08862605231190340 (PMC10619170; doi:10.1177/08862605231190340)
Supplement: sj-docx-1-jiv-10.1177_08862605231190340 – Supplemental material for Perpetrator Perceptions on the Emotions and Motivations Driving Technology-Facilitated Abuse in Relationships: A Story Completion Study [file sj-docx-1-jiv-10.1177_08862605231190340.docx]

**Supplementary material - Story Stems Provided to Participants**

| **Story Number** | **Story Type/Story Stem** |
| --- | --- |
|  | **Story Stem Presented to All Participants** |
| 1 | Chris and Alex are having trouble in their relationship, and it has been weighing on Chris’ mind. Alex is upstairs asleep, and Chris is awake and worrying about the relationship. Chris notices that Alex’s phone and laptop are accessible on the kitchen table and….  Instruction: *Please write about what Chris thinks, does and feels, and why Chris chooses that course of action.* |
|  | **Story Stems Randomly Present to Participants** |
| 2 | Sam and Ash have recently split, and Sam is pretty upset about the situation still. Sam begins scrolling through photos and videos the two of them took together, some of which are quite explicit, and….  Instruction: *Please write about what Sam thinks, feels and does, and why Sam chooses that course of action.* |
| 3 | Sam and Ash have been having trouble in their relationship recently. Sam expected Ash home two hours ago, and Ash hasn’t been in touch. Sam opens a laptop and…  Instruction: *Please write about what Sam thinks, feels and does, and why Sam chooses that course of action.* |
| 4 | Sam and Ash need new phones. Sam offers to buy them and set them up. Ash agrees, so Sam buys the phones and when setting them up Sam….  Instruction: *Please write about how Sam sets the phones up, why they are set up the way they are, and how Sam feels about it.* |
| 5 | Sam and Ash have recently split, and Ash contacts Sam to ask that the videos that they took of their intimate activities in happier times be deleted. On the mobile phone, Sam…  Instruction: *Please write about what Sam thinks, feels and does, and why Sam chooses that course of action.* |
| 6 | Sam and Ash have recently split. Sam hears through friends that Ash has a new partner. Sam logs onto the internet and …  Instruction: *Please write about what Sam thinks, feels and does, and why Sam chooses that course of action.* |
| 7 | Sam notices that in the past three weeks Ash has been spending more on clothes —new shoes, pants and tops. Ash came home again today with three new pairs of shoes and a new pair of jeans. After Ash goes to bed, Sam grabs a phone or laptop and …  Instruction: *Please write about what Sam thinks, feels and does, and why Sam chooses that course of action.* |
| 8 | Sam and Ash are building a new home together, and they need to decide on home automation technologies, Ash says to Sam ‘you know I don ’t know about any of this stuff, you can decide’. Sam chooses…  Instruction: *What home automation does Sam choose, and why?* |
